# Supplementary material for: Responsive Alternative Splicing Events of Opisthopappus Species against Salt Stress
Source: Int J Mol Sci. 2024 Jan 19;25(2):1227. doi: 10.3390/ijms25021227 (PMC10816081; doi:10.3390/ijms25021227)
Supplement: Supplementary file 1 [file ijms-25-01227-s001.zip › ijms-2731539-supplementary.pdf]

Supplementary Materials

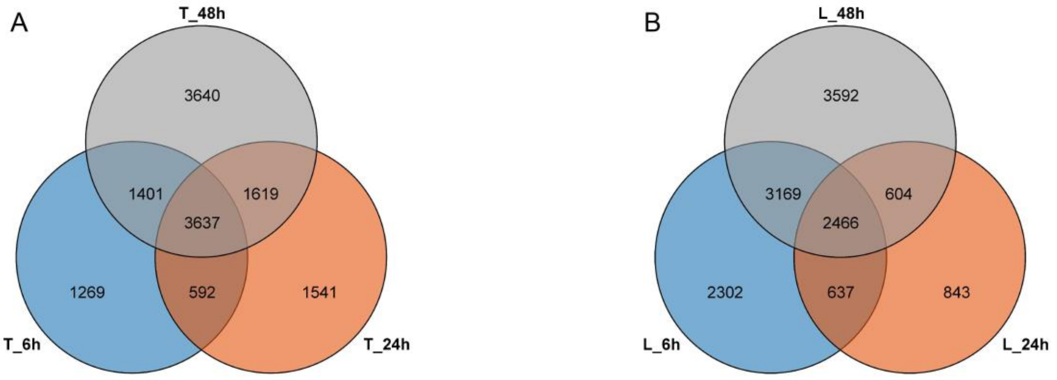

Figure S1. Venn diagram analysis showing DEGs under different time treatments. (A) DEGs of *O. taihangensis.* (B) DEGs of *O. longilobus.*

Note: T: *O. taihangensis*. L: *O. longilobus*.

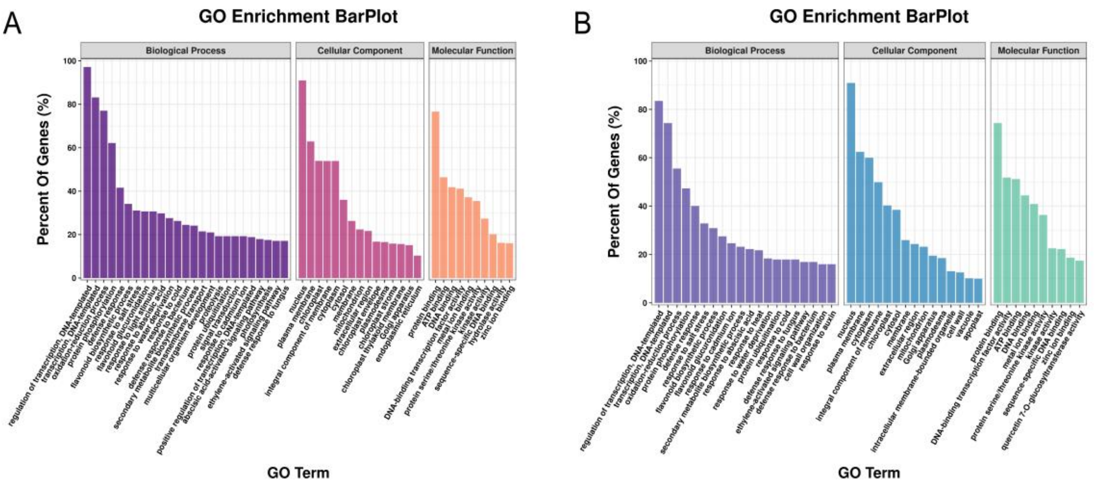

Figure S2. GO enrichment analysis of shared DEGs under time gradients. (A) GO enrichment analysis of *O. taihangensis.* (B) GO enrichment analysis of *O. longilobus.*

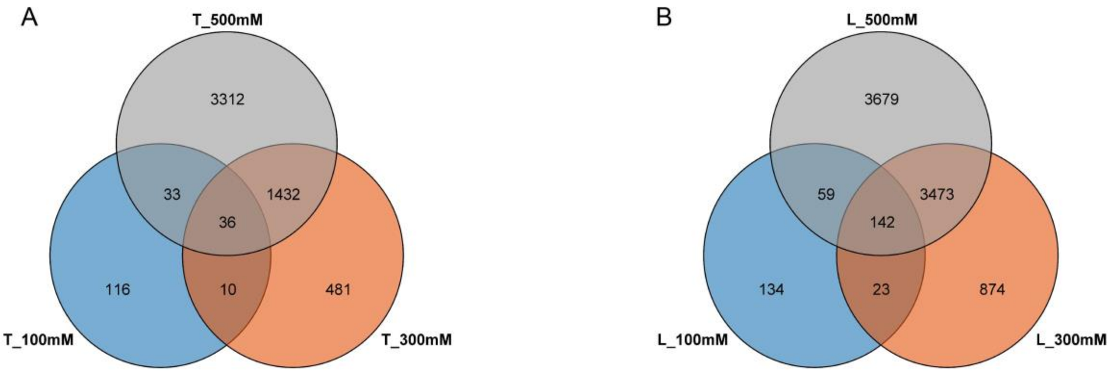

Figure S3. Venn diagram analysis showing DEGs under different concentration gradients. (A) DEGs of *O. taihangensis.* (B) DEGs of *O. longilobus.*

Note: T: *O. taihangensis*. L: *O. longilobus*.

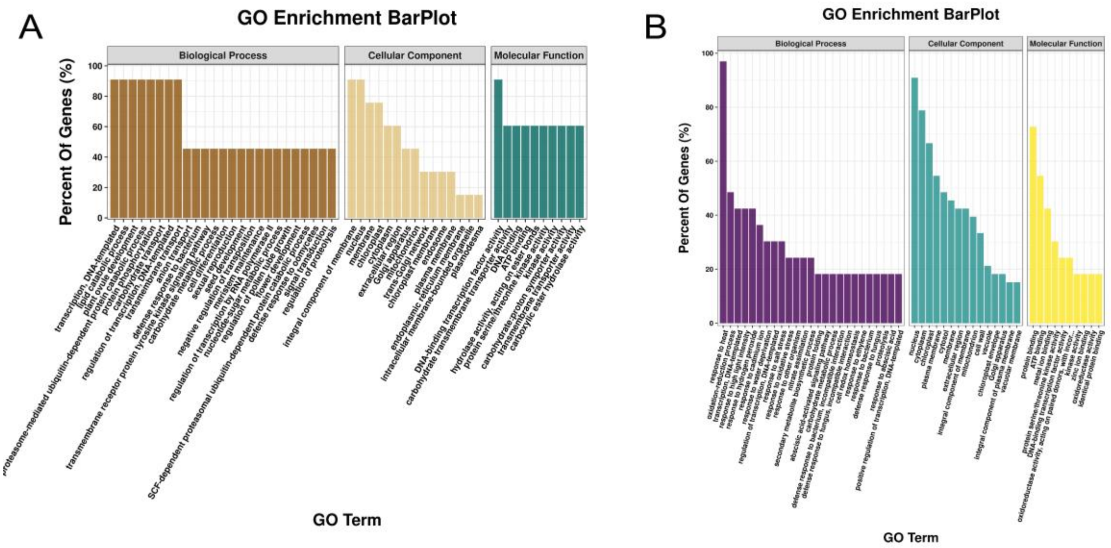

Figure S4. GO enrichment analysis of shared DEGs under concentration gradients. (A) GO enrichment analysis of *O. taihangensis.* (B) GO enrichment analysis of *O. longilobus.*

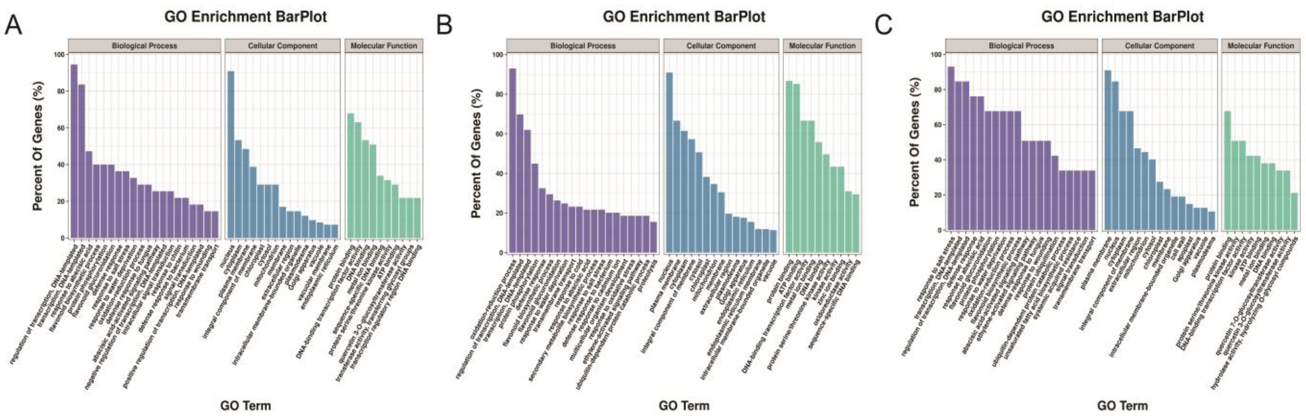

Figure S5. GO enrichment analysis of DEGs in three classes. (A) GO enrichment analysis of DEGs in class one. (B) GO enrichment analysis of DEGs in class two. (C) GO enrichment analysis of DEGs in class three.

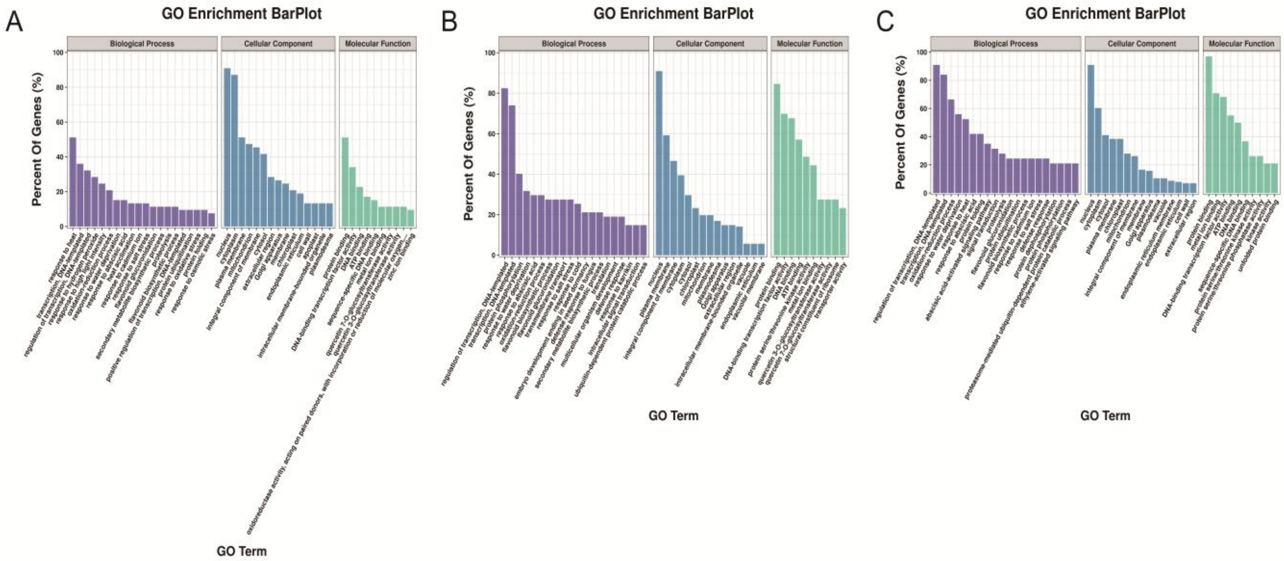

Figure S6. GO enrichment analysis of DEGs in three classes. (A) GO enrichment analysis of DEGs in class one. (B) GO enrichment analysis of DEGs in class two. (C) GO enrichment analysis of DEGs in class three.

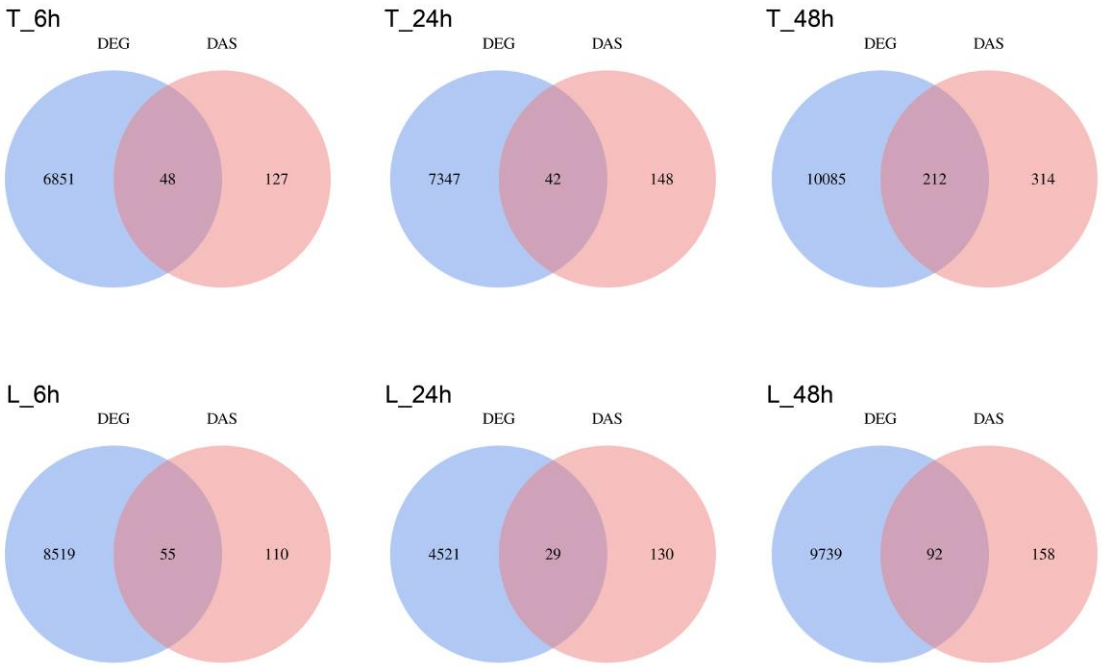

Figure S7. Venn diagram analysis showing overlaps between DEG and DAS under time gradients.

Note: T: *O. taihangensis*. L: *O. longilobus*.

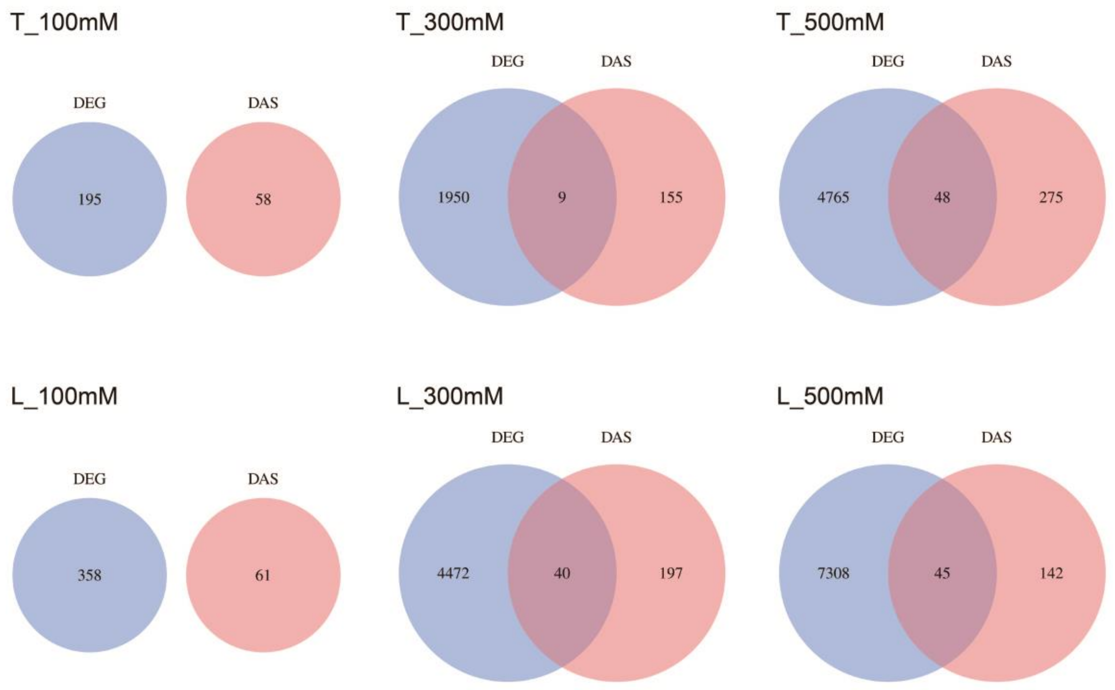

Figure S8. Venn diagram analysis showing overlaps between DEG and DAS under concentration gradients.

Note: T: *O. taihangensis*. L: *O. longilobus*.

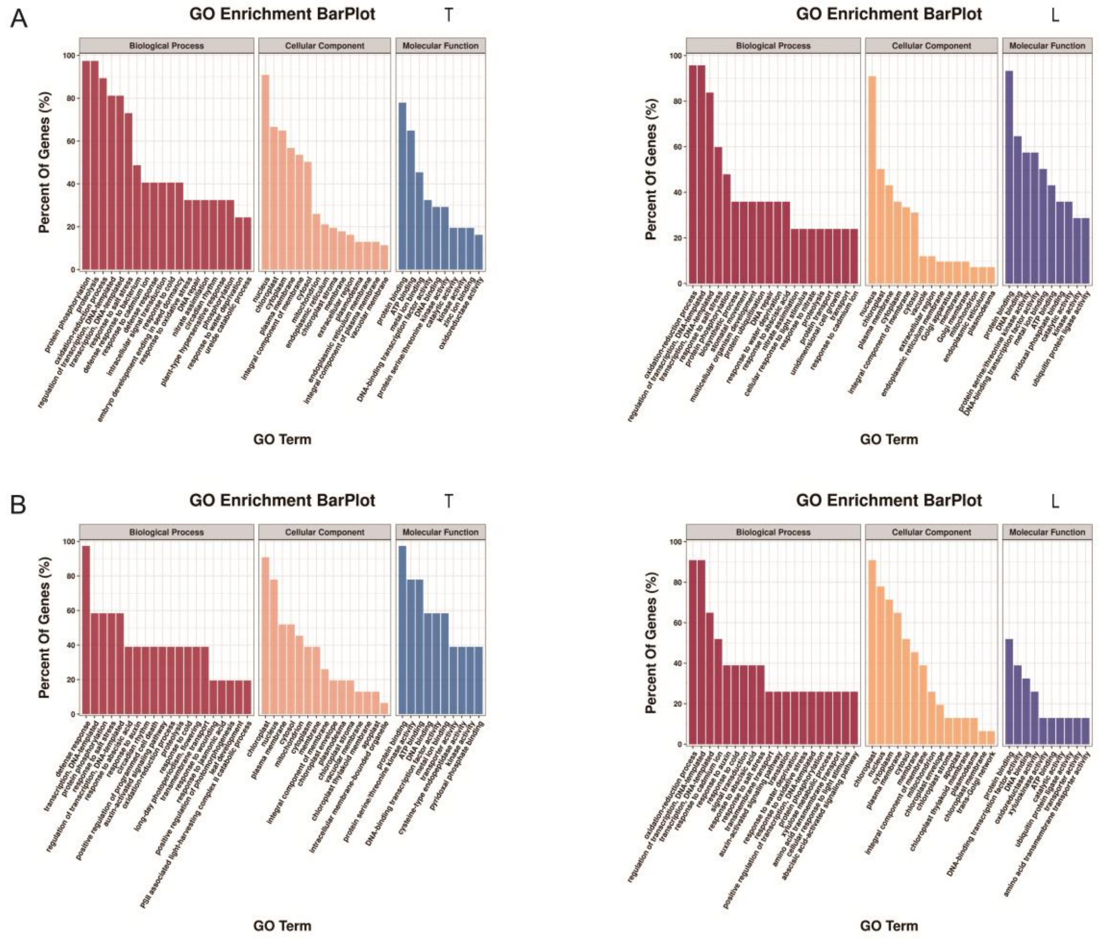

Figure S9. GO enrichment analysis of overlapped genes. (A) GO enrichment analysis of overlapped genes under time gradients. (B) GO enrichment analysis of overlapped genes under concentration gradients.

Note: T: *O. taihangensis*. L: *O. longilobus*.

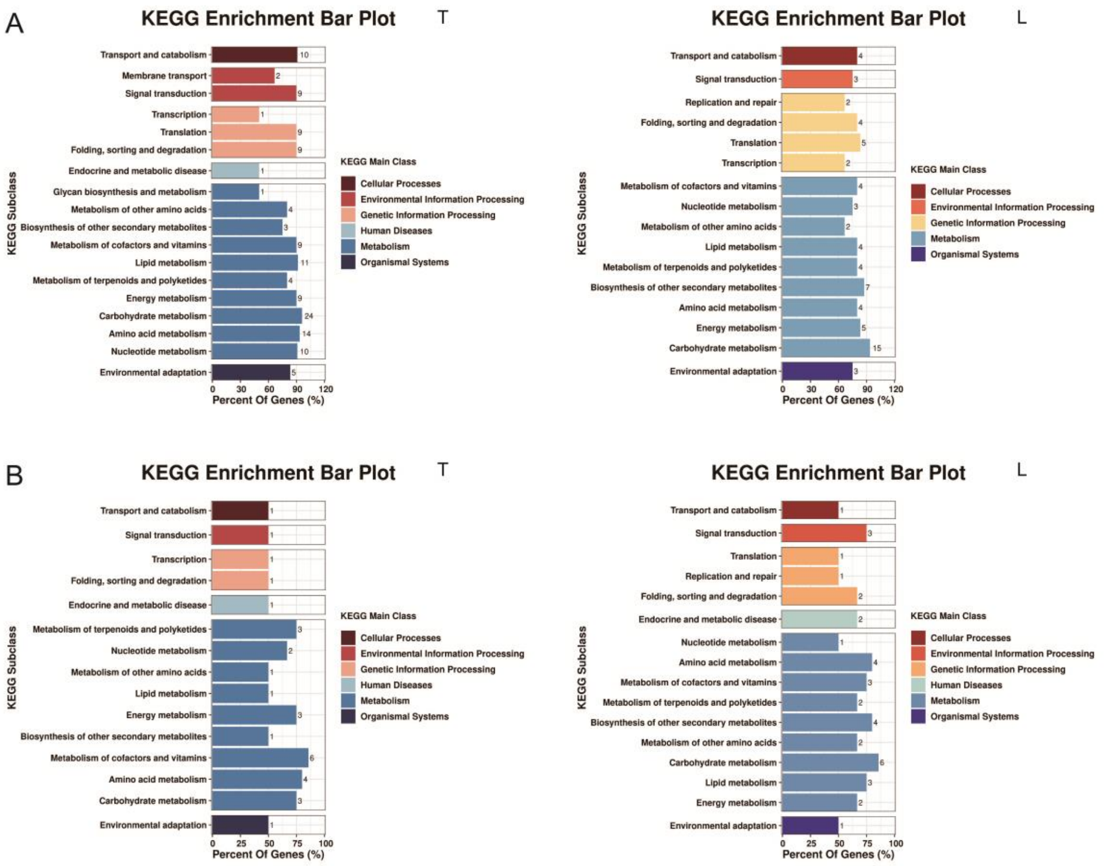

Figure S10. KEGG enrichment analysis of overlapped genes. (A) KEGG enrichment analysis of overlapped genes under time gradients. (B) KEGG enrichment analysis of overlapped genes under concentration gradients.

Note: T: *O. taihangensis*. L: *O. longilobus*.

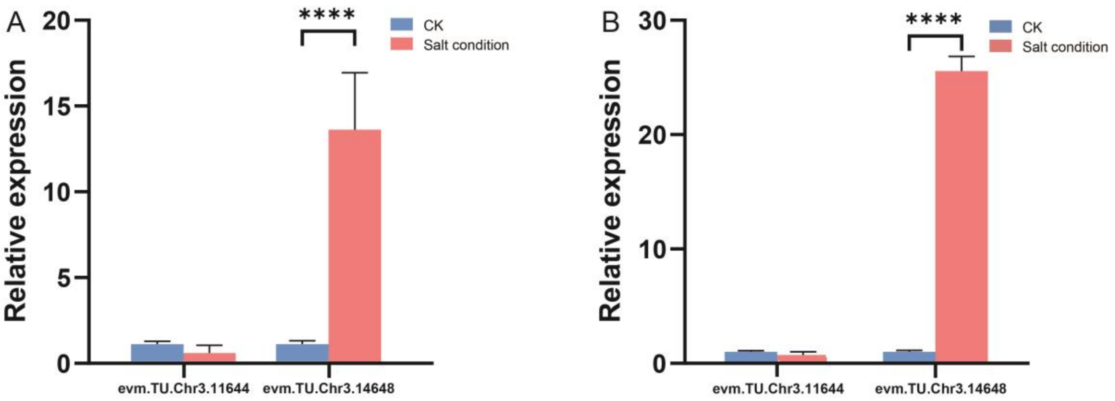

Figure S11. Experimental validation for DAS events under time gradients. (A) qRT-PCR analysis of *O. taihangensis.* (B) qRT-PCR analysis of *O. longilobus.*

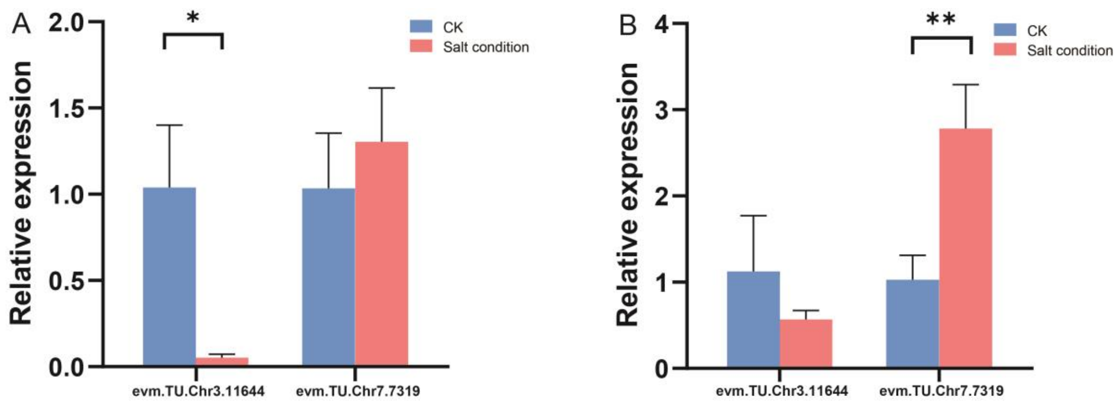

Figure S12. Experimental validation for DAS events under concentration gradients. (A) qRT-PCR analysis of *O. taihangensis.* (B) qRT-PCR analysis of *O. longilobus.*

Table S1. Primers for qRT-PCR validation.

Gene ID	Primers (5'-3')
evm.TU.Chr3.11644	F: TCATTCTCAGAGCCTCACAAGC<br>R: TGGCACTAGAACCAACCATACC
evm.TU.Chr3.14648	F: ATGCTCGTCTTCAGGTTCTCA<br>R: CCAAGGACACTGTTGTGATTCT
evm.TU.Chr7.7319	F: AGCGGTATCAGTCCTTAGATGG<br>R: CTGCGGTCTTCGTTCCTATGT
evm.TU.Chr8.13443 (Actin)	F: CCTACAACGCCACACTCTCA<br>R: ACAGCAAGTTACACCACTCATG

Table S2. DEGs under different salt treatments

Sample	Number of DEGs	Up	Down
T_6h	6899	3986	2913
T_24h	7389	4174	3215
T_48h	10297	5504	4793
L_6h	8574	4483	4091
L_24h	4550	2764	1786
L_48h	9831	5425	4406
T_100mM	195	77	118
T_300mM	1959	1127	832
T_500mM	4813	2593	2220
L_100mM	358	189	169

L_300mM	4512	2333	2179
L_500mM	7353	3767	3586

Note: T: *O. taihangensis*. L: *O. longilobus*.

Table S3. Statistics on the number of AS events

Sample	Number of AS events	SE	MXE
T_6h	4721	4555	166
T_24h	4831	4647	184
T_48h	4324	4180	144
L_6h	5271	5060	211
L_24h	5387	5148	239
L_48h	4428	4274	154
T_100mM	4294	4122	172
T_300mM	5154	4945	209
T_500mM	6660	6366	294
L_100mM	3941	3796	145
L_300mM	5495	5262	233
L_500mM	5311	5089	222

Note: T: *O. taihangensis*. L: *O. longilobus*.

Table S4. Statistics on the number of DAS events

Sample	Number of DAS events	SE	MXE
T_6h	180	173	7
T_24h	198	194	4
T_48h	552	529	23
L_6h	175	173	2
L_24h	162	156	6
L_48h	258	242	16
T_100mM	60	57	3
T_300mM	172	163	9

T_500mM	334	324	10
L_100mM	62	62	0
L_300mM	242	241	1
L_500mM	190	182	8

Note: T: *O. taihangensis*. L: *O. longilobus*.
